# Prognostic Implications of the Number of Lymph Node Metastases in Oral Tongue Squamous Cell Carcinoma: A Population Study of the SEER Database and an Institutional Registry

**DOI:** 10.1002/cam4.70493

**Published:** 2024-12-19

**Authors:** Wenjie Huang, Yu Zhang, Hao Li, Zhiying Liang, Shumin Zhou, Jie Pan, Hui Xie, Chao Luo, Shuqi Li, Guangying Ruan, Fei Ai, Yanfeng Chen

**Affiliations:** ^1^ State Key Laboratory of Oncology in South China Collaborative Innovation Center for Cancer Medicine, Sun Yat‐Sen University Cancer Center Guangzhou China; ^2^ Department of Radiology Sun Yat‐Sen University Cancer Center Guangzhou China; ^3^ Department of Pathology Sun Yat‐Sen University Cancer Center Guangzhou China; ^4^ Department of Oral & Maxillofacial Surgery Sun Yat‐Sen University Cancer Center Guangzhou China

**Keywords:** AJCC, lymph node metastasis, oral tongue, squamous cell carcinoma, TNM staging

## Abstract

**Background:**

To investigate the impact of the number of positive lymph nodes (PLNs) on long‐term survival and pathological nodal stage in patients with oral tongue squamous cell carcinoma (OTSCC).

**Materials and Methods:**

Newly diagnosed and nonmetastatic adult patients with OTSCC who underwent curative resection were identified between January 2010 and December 2020. External validation was performed via the SEER registry. Multivariate Cox proportional hazards model was employed to calculate hazard ratios (HRs) and 95% confidence intervals (CIs) of pathological nodal features. Propensity score matching (PSM) was used to assess effect of adjuvant chemoradiotherapy (ACRT).

**Results:**

Among 518 curative‐intent OTSCC patients, the number of PLNs independently predicted overall survival (OS), surpassing other pathological nodal features, including extranodal extension, laterality, and lower neck involvement. Patients with 1 or 2 PLNs had comparable worse OS than those with no PLN (median OS of 1 PLN vs. 2 PLNs vs. 0 PLN: 35.1 vs. 30.5 vs. 40.2 months), but better than those with ≥ 3 PLNs (median OS of 1–2 PLNs vs. 3 PLNs: 32.1 vs. 19.0 months). A proposed nodal category with 0, 1–2 PLNs, and ≥ 3 PLNs exhibited increasingly worse OS (HR of 1–2 PLNs and ≥ 3 PLNs vs. 0 PLN: 2.98 [95% CI: 1.89–4.71], *p* < 0.001; 5.47 [95% CI: 3.33–9], *p* < 0.001; respectively) and showed improved prediction power versus current pN staging (C‐index: 0.717 vs. 0.713, *p* < 0.001). PSM analysis revealed that ACRT benefited patients with advanced nodal disease (≥ 3 PLNs) and improved OS. These findings were validated in SEER registry.

**Conclusion:**

The number of PLNs is a better predictor of overall tumor burden for OTSCC and could be a more accurate metric for survival estimation, which should be considered in future simplified pathological nodal staging for better risk stratification and decision‐making in subsites of the oral cavity.

## 1 Introduction

Squamous cell carcinoma (SCC) of the head and neck ranks as the seventh most common cancer worldwide [1]. SCC of the oral tongue (OTSCC) is biologically distinct from cancers arising in other subsites of the head and neck and is characterized by non‐HPV‐associated cancer [2], increasing incidence [3], and a more aggressive clinical presentation [4, 5]. Although the therapeutic practice has evolved, with chemotherapy, irradiation, and immunotherapy now available in the curative protocol, glossectomy combined with neck resection remains the cornerstone of treatment. Unfortunately, tumor control remains unsatisfactory, with 5‐year overall survival (OS) rates ranging from approximately 30% to 66% among patients treated at high‐volume centers for curative‐intent resection of OTSCC [6, 7]. These findings highlight the need for greater focus on the OTSCC subtype.

Lymph node metastasis is a poor prognostic factor in head and neck SCC and other solid tumors [8, 9, 10]. Patients with even a single metastatic lymph node are classified as having advanced disease, with a 50% decrease in OS. The current American Joint Committee on Cancer/International Union Against Cancer (AJCC/UICC) N‐staging system for oral cancers serves as a prognostic tool and includes size, laterality, extranodal extension (ENE), and the number of positive lymph nodes (PLNs) [11]. However, certain limitations persist in N staging [12, 13, 14], including the complex nodal staging schema, overlapping survival curves within the pN1‐N3b categories, and the narrow scopes of specific subgroups, consequently impacting its prognostic capability. For example, the pN2a status is only for patients with 1 PLN that is less than 3 cm across, whereas the pN3a status is reserved for patients with a node size greater than 6 cm in the absence of ENE. Moreover, applying a single nodal schema to oral entities with heterogeneous biological behaviors based on distinct anatomical backgrounds may not be adequate. Therefore, proposing a simplified and practical nodal category specific to OTSCC is important for precise survival estimation and treatment management.

Additionally, two large‐scale randomized trials, RTOG 95–012 [15] and EORTC 22931 [16], demonstrated the advantages of adjuvant chemoradiotherapy (ACRT) over adjuvant radiotherapy (RT) for patients with high‐risk head and neck cancers. These studies identified positive margins and ENE as appropriate criteria for adding chemotherapy. Despite numerous studies highlighting the benefits of concurrent ACRT over RT alone in head and neck cancers, data specifically addressing the survival benefit of ACRT in patients with OTSCC remain scarce. Therefore, determining the optimal lymph node metastatic burden threshold for OTSCC patients to benefit from ACRT in terms of survival is a further question that needs to be addressed. In this study, we evaluated the prognostic and therapeutic impact of the number of pathological PLNs among OTSCC patients at the institutional level with detailed clinicopathological data and applied the Surveillance, Epidemiology, and End Results (SEER) registry for external validation.

## 2 Material and Methods

### 2.1 Study Cohort and Ethics

**FIGURE 1:**
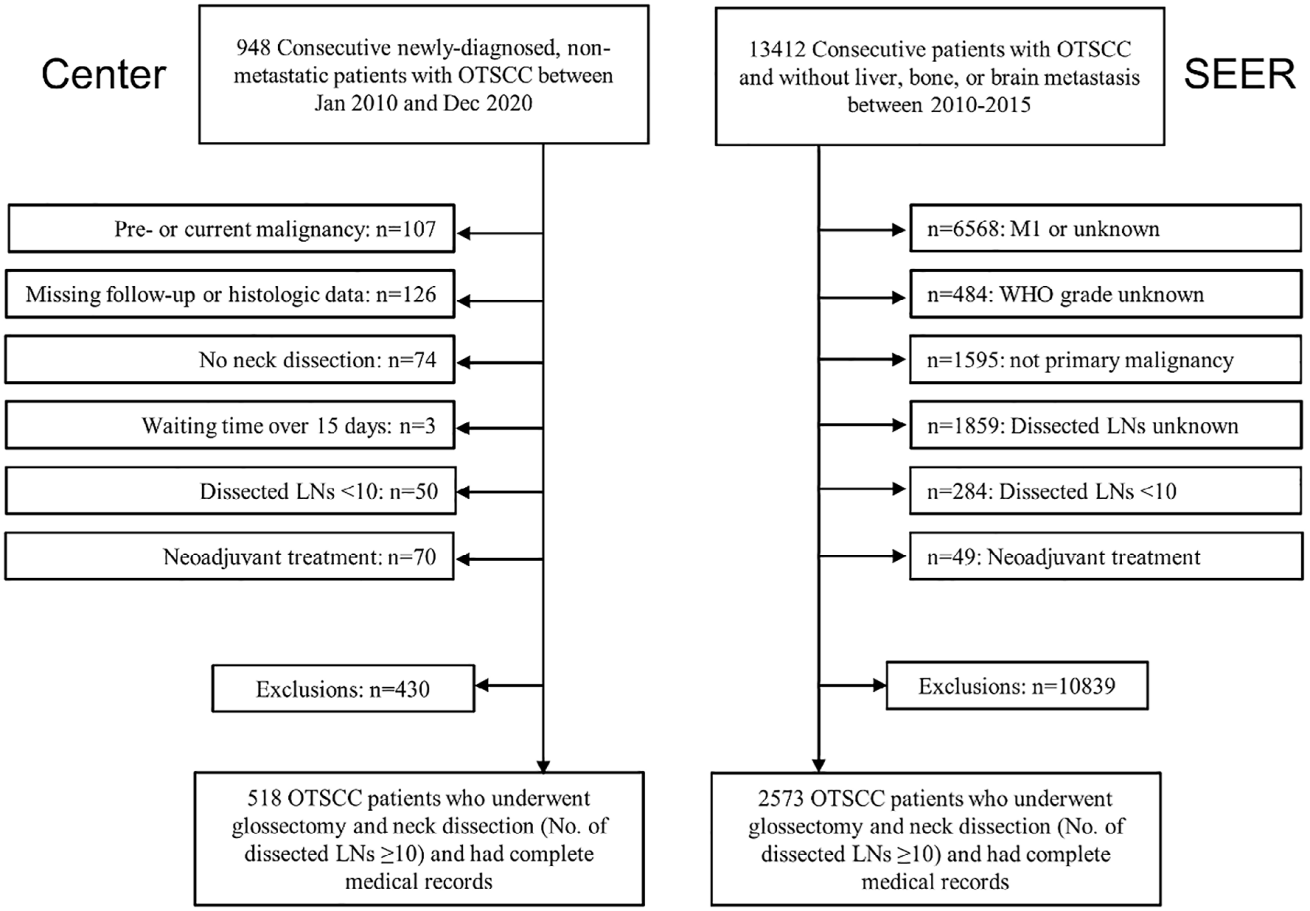
Flowchart of patient selection. OTSCC, oral tongue squamous cell carcinoma; SEER, Surveillance, Epidemiology, and End Results; LNs, lymph nodes.

We identified 948 consecutive adult patients with newly diagnosed, nonmetastatic OTSCC who underwent curative surgical treatment from January 2010 to December 2020. Among them, 430 were excluded due to prior/synchronous cancers (*n* = 107), incomplete histologic data/follow‐up (*n* = 126), no neck dissection (*n* = 74), > 15 days before surgery (*n* = 3), < 10 examined lymph nodes (*n* = 50), or neoadjuvant treatment (*n* = 70) (Figure 1). This study aimed to determine the significance of the pathological nodal characteristics of PLNs to optimize N staging. We specifically selected patients with at least ≥ 10 dissected lymph nodes to ensure precise pathological N staging as per the 8th edition of the AJCC/UICC guidelines. After applying the exclusion criteria, a total of 50 patients were excluded from further analysis. All patients were restaged according to the 8th AJCC TNM staging system [11]. Treatment adhered to University Cancer Center guidelines (Appendix S1). This retrospective study was conducted in accordance with the 1964 Helsinki Declaration. The study design was approved by the ethics committee (approval number B2019‐047‐Y02) on November 07, 2022. Our research adhered to the STROCSS criteria16 for reporting [17].

### 2.2 Follow‐Up and Endpoints

The follow‐up frequency was at least once every 1–3 months during the first year and then once every 3–12 months thereafter. Follow‐up data up to December 2022 were collected via telephone or outpatient visits. The main clinical endpoint in this study was OS, defined as the period between the date of initial treatment and the date of death from any cause or censoring at the date of the last follow‐up. Disease‐free survival (DFS) was calculated from the date of surgery to the date of disease relapse, distant metastasis, or death from any cause, and locoregional‐free survival (LRFS) was defined as the date of surgery to the date of disease relapse.

### 2.3 Data Collection From the SEER Registry

Patients who underwent curative resection without brain, liver, or bone metastasis were identified in the SEER database between 2010 and 2015 via the 3rd edition of the International Classification of Disease for Oncology‐3 codes. Patient data were collected via the primary site code for the dorsal surface of the tongue (C02.0), border of the tongue (C02.1), ventral surface of the tongue (C02.2), anterior of the tongue (C02.3), overlapping lesions of the tongue (C02.8), and tongue (C02.9), as well as the histology code for squamous cells (8050–8089). Among the 13,412 OTSCC patients identified, those with 7th edition AJCC M1/unknown stage (*n* = 6568), unknown histological grade (*n* = 484), non‐primary tongue lesions (*n* = 1595), < 10/unknown dissected lymph nodes (*n* = 2143), and neoadjuvant treatment (*n* = 49) were excluded. The final validation cohort included 2573 patients (Figure 1).

### 2.4 Statistical Analysis

Categorical variables are described via frequency rates and percentages, whereas continuous data are presented as medians and interquartile ranges (IQRs). The reverse Kaplan–Meier method was used to calculate the median follow‐up [18]. Survival estimations were determined via the Kaplan–Meier method, and differences were compared via the log‐rank test. Univariate and multivariate analyses were conducted via Cox regression proportional hazards models, and hazard ratios (HRs) with corresponding 95% confidence intervals (CIs) were estimated. Non‐nodal‐related clinicopathological variables with a *p*‐value < 0.05 in the univariate analysis were further included in the multivariate analysis for determining confounding factors. The optimal cutoff value was identified by analyzing the frequency distribution of metastatic lymph nodes [19], with subgroups exhibiting similar prognoses combined to optimize risk stratification [20]. Harrell's concordance index (C‐index) and its 95% CI were applied to evaluate the discrimination ability of the proposed nodal category and were used to compare the robustness between this system and the 8th AJCC pN‐staging system. Propensity score matching (PSM) was used to minimize selection bias and create a balance between baseline variables. Patients with and without ACRT after curative resection were matched via nearest‐neighbor matching (1:1) and a 0.2 caliper width. Because age was a categorical variable in the SEER cohort, age in our center cohort was categorized with a cutoff value of 45 for PSM analyses [21]. Statistical analyses were performed via the R package (Version 4.2.2; mice, rms, survival, SiZer, party libraries), with the two‐sided significance level set at 0.05.

## 3 Results

There were 518 cases of OTSCC in the institutional study population, comprising 349 men and 169 women. The median age was 52 years (range, 19–82 years). The median number of dissected lymph nodes was 20 (range, 10–89). At the time of surgical resection, 184 (35.5%) patients had one or more PLNs. During the postoperative period, 73 (39.7%) patients with at least 1 PLN and 23 (6.9%) patients without nodal disease received ACRT. After a median follow‐up of 44.8 months (95% CI, 42–47.5 months), 120 (23.2%) patients died, whereas 153 (29.5%) and 86 (16.6%) patients experienced disease progression and relapse, respectively. The overall median, 1‐, 3‐, and 5‐year OS rates were 36.8 months, 81.9%, 78.3%, and 73.3%, respectively.

### 3.1 The Impact of the Number of Positive Lymph Nodes on Long‐Term Survival

On multivariate analysis, age, diabetes, betel nut chewing, pathological differentiation, lymphovascular invasion, and pT stage independently predicted OS (all *p* < 0.05) and were selected as confounding factors for adjusting the impact of the nodal features (Table S1).

**FIGURE 2:**
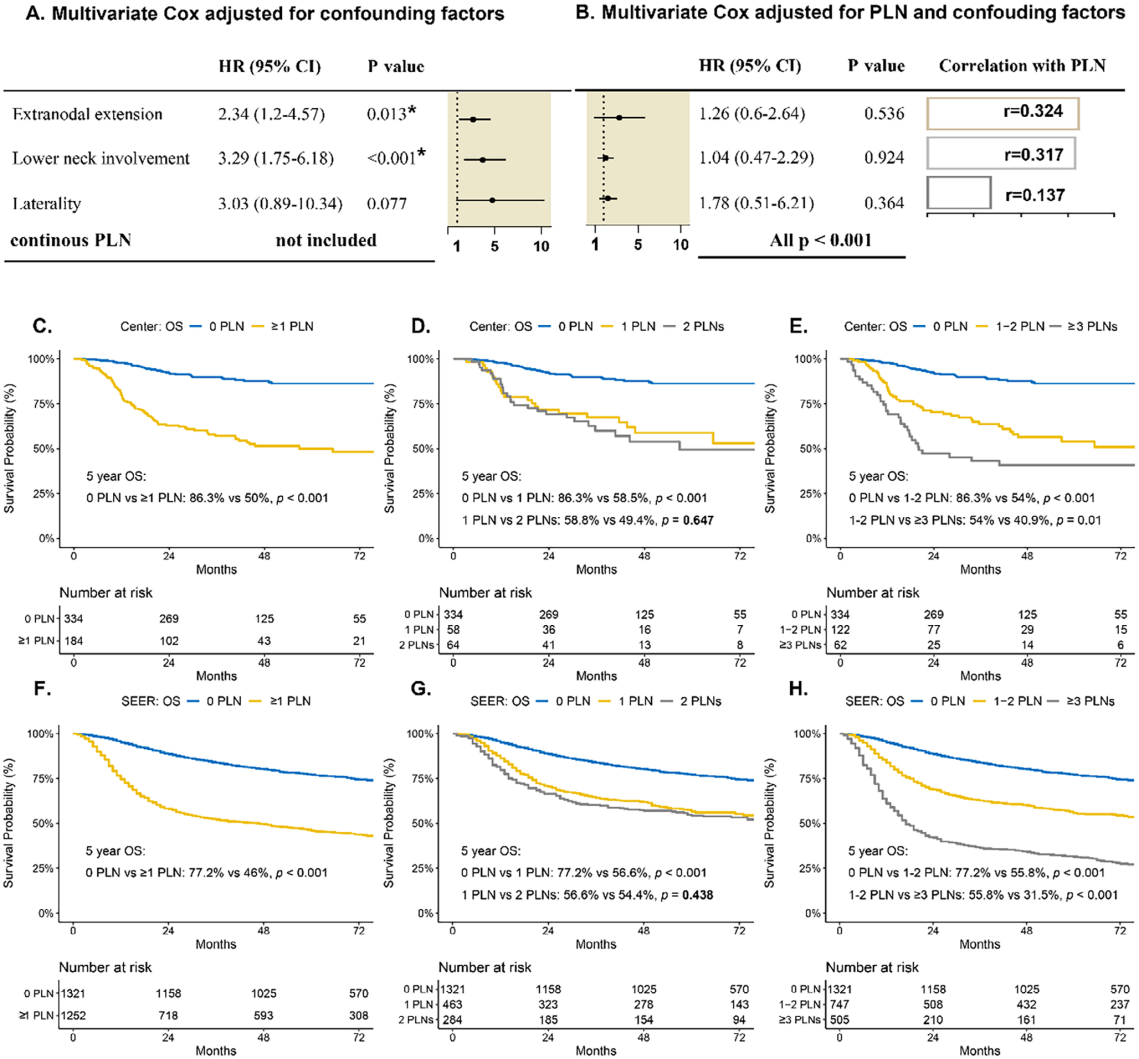
Forest plot and Kaplan–Meier analysis of overall survival by the PLN. (A) After adjusting for confounding factors (age, diabetes, betel nut chewing, differentiation, pT stage, and lymphovascular invasion), extranodal extension (ENE), and lower‐neck involvement were independent predictors of overall survival (OS), while laterality marginally approached significant. (B) After adjusting for the continuous number of PLNs and the same confounding factors, ENE, lower neck involvement, and laterality were not independent predictors of OS, but in each multivariate analysis, the number of PLNs was the independent predictor of OS. (C–H) A significant difference was observed between the patients with no nodal disease versus nodal disease, yet no significant difference was observed between the patients with one PLN versus those with two PLNs. Thus, we combined patients with one or two PLNs as one group. CI, confidence interval; HR, hazard ratio; OS, overall survival; PLN, positive lymph node; SEER, Surveillance, Epidemiology, and End Results Program.

According to the univariable analyses, the number of PLNs was strongly negatively associated with OS (*p* < 0.001; Table 1). In the adjusted multivariate Cox regression model without the number of PLNs, the presence of ENE (*p* = 0.013) and lower neck involvement (*p* < 0. 001) independently predicted 5‐year OS, whereas laterality approached significance (*p* = 0. 077) (Figure 2A). In contrast, when the model incorporated the number of PLNs, neither the presence of ENE, lower neck involvement, nor laterality independently predicted OS (Figure 2B). When the number of PLNs was considered along with ENE, lower neck involvement, and laterality, PLNs remained strongly associated with OS, and its significance still surpassed that of other adverse nodal pathological factors (Table S2).

**TABLE 1:** Characteristics of patients with OTSCC.

Variables	Total (*N* = 518)	5‐years (%)	*p*
Age (years)	52 (43–61)	—	0.014
Sex			
Male	349 (67.4%)	70.72	0.075
Female	169 (32.6%)	78.4	
BMI (kg/m^2^)	22.5 (20.5–24.8)	—	0.313
Smoking			
No	338 (65.3%)	75.7	0.025
Yes	180 (34.7%)	68.78	
Alcohol use			
No	426 (82.2%)	74.06	0.158
Yes	92 (17.8%)	69.89	
Betel net			
No	484 (93.4%)	75.03	0.004
Yes	34 (6.6%)	37.91	
Hypertension			
No	431 (83.2%)	73.2	0.399
Yes	87 (16.8%)	74.2	
Diabetes			
No	471 (90.9%)	74.66	0.008
Yes	47 (9.1%)	59.34	
Family history			
No	478 (92.3%)	73.98	0.276
Yes	40 (7.7%)	65.02	
Differentiation			
Well	307 (59.3%)	68.51	0.018
Moderate‐poor	211 (40.7%)	79.51	
Lymphovascular involvement			
No	495 (95.6%)	74.35	0.006
Yes	23 (4.4%)	50.72	
Perineural invasion			
No	333 (64.3%)	79.59	< 0.001
Yes	185 (35.7%)	60.49	
Margin status			
Negative	513 (99%)	73.92	0.106
Positive	5 (1%)	40	
pT stage			
T1	156 (30.1%)	89.41	< 0.001
T2	188 (36.3%)	73.88	
T3	164 (31.7%)	57.08	
T4	10 (1.9%)	28.57	
pN stage			
N0	334 (64.5%)	86.32	< 0.001
N1	57 (11%)	59.86	
N2	106 (20.5%)	45.93	
N3	21 (4.1%)	42.49	
ACRT			
No	422 (81.5%)	75.5	0.011
Yes	96 (18.5%)	63.02	
Neck dissection			
Laterality	491 (94.8%)	73.87	0.078
Bilaterality	27 (5.2%)	64.96	
No. of positive lymph nodes	0 (0–1)	—	< 0.001
No. of dissected lymph nodes	20 (15–26)	—	0.122
Lower neck involvement			
No	499 (96.3%)	74.86	< 0.001
Yes	19 (3.7%)	30.62	
ENE			
Negative	495 (95.6%)	74.53	< 0.001
Positive	23 (4.4%)	42.29	
Laterality (N2c)			
Unilaterality	514 (99.2%)	73.79	0.001
Bilaterality	4 (0.8%)	NA	

*Note:* Continuous data are medians, with IQRs in parentheses.

Abbreviations: ACRT, adjuvant chemoradiotherapy; BMI, body mass index; ENE, extranodal extension; IQR, interquartile ranges; NA, not available; OTSCC, oral tongue squamous cell carcinoma.

### 3.2 Impact of the Number of Positive Lymph Nodes on Nodal Staging

The number of PLNs is a better predictor of overall tumor burden than other variables and could be a more accurate metric for nodal staging. Patients with nodal involvement had a worse OS than those without nodal involvement (median OS: 28.2 months for those with ≥ 1 PLN vs. 40.2 months for those with 0 PLN, *p* < 0.001) (Figure 2C). The distribution of PLNs is detailed in Figure S1, with a marked decline in patient numbers beyond three PLNs. Stratification by PLN count revealed prognostic differences: patients with one or two PLNs had a worse OS than patients with no nodal disease (median OS: 35.1 months for one PLN, 30.5 months for two PLNs, and 40.2 months for 0 PLN) (Figure 2D), and patients with three or more PLNs had the poorest prognosis (median OS: 19.0 months for ≥ 3 PLNs vs. 32.1 months for 1–2 PLNs, *p* = 0.01) (Figure 2E). Similar results were observed for DFS (median OS: 35.6 months for 0 PLN, 26.7 months for 1–2 PLNs, and 10.7 months for ≥ 3 PLNs; all *p* < 0.05) and LRFS (35.9 months vs. 30.5 months vs. 17.3 months; all *p* < 0.05) (Figure S2). For the SEER population, patients with 0, 1–2, and ≥ 3 PLNs had increasingly worse OS (median OS: 66.0 months for 0 PLN, 54.0 months for 1–2 PLNs, and 17.0 months for ≥ 3 PLNs; all *p* < 0.001) (Figure 2F–H).

A simplified nodal categorization consisting of the number of PLNs was developed (0 PLN, 1–2 PLNs, and ≥ 3 PLNs). In the adjusted multivariate analysis, the proposed nodal category independently predicted OS, with a 5‐year OS of 86.3% for the 0‐PLN group, 54% for the 1–2‐PLNs group (HR: 2.98 [1.89, 4.71]; *p* < 0.001), and 40.9% for the ≥ 3‐PLNs group (HR: 5.47 [3.33, 9]; *p* < 0.001) (Table 2). The C‐index for the proposed nodal category showed better predictive ability (0.717, 95% CI [0.673 to 0.761]) than did the 8th edition AJCC N‐staging (0.713, 95% CI [0.669 to 0.757]) (*p* = 0.005), which was validated in the SEER registry (0.674, 95% CI [0.659–0.69] vs. 0.666, 95% CI [0.65 to 0.681]; *p* < 0.001).

**TABLE 2:** Multivariate Cox regression analyses for patients with OTSCC regarding overall survival.

Variables	HR (95% CI)	*p*
Age	1.02 (1, 1.03)	0.015
Betel nut		
No		
Yes	2.72 (1.48, 5)	0.001
Diabetes		
No		
Yes	1.94 (1.13, 3.32)	0.016
pT stage		
T1		
T2	1.96 (1.08, 3.58)	0.027
T3	2.72 (1.52, 4.87)	0.001
T4	2.23 (0.72, 6.92)	0.164
Differentiation		
Well		
Moderate‐poor	0.74 (0.5, 1.11)	0.142
Lymphovascular involvement		
No		
Yes	1.57 (0.78, 3.19)	0.208
Simplified nodal category		
0 PLN		
1–2 PLNs	2.98 (1.89, 4.71)	< 0.001
≥ 3 PLNs	5.47 (3.33, 9)	< 0.001

Abbreviations: CI, confidence interval; HR, hazard ratio; OTSCC, oral tongue squamous cell carcinoma; PLN, positive lymph node.

Given the similar prognoses of patients with one PLN and two PLNs, we further divided the 59 patients with current pN2 and 1–2 PLNs into pN1, with descending stage shifts of pN1 57 (11%) and pN2 106 (20.5%) to pN1 116 (22.4%) and pN2 47 (9.1%). After downstaging current pN2 with 1–2 PLNs to pN1, the survival curves of pN1 and pN2 were separated (before adjustment, *p* = 0.088; after adjustment, *p* = 0.015), with a significantly improved C‐index of the whole pN stage (C‐index before vs. after adjustment, 0.713 95% CI [0.669 to 0.757] vs. 0.716 95% CI [0.673 to 0.76], *p* = 0.004). In the SEER registry, a total of 253 patients with pN2 and 1–2 PLNs were downstaged to pN1, also showing a decreasing sample size of pN1 532 (20.7%) and pN2 704 (27.4%) to pN1 785 (30.5%) and pN2 451 (17.5%) and a significantly improved C‐index (0.673 95% CI [0.658 to 0.689] vs. 0.666 95% CI [0.65 to 0.681], *p* < 0.001). These findings underscore the importance of considering a stratified number of PLNs (1 to 2) in future optimizations of pN staging.

### 3.3 The Impact of the Proposed Nodal Category on Adjuvant Treatment

**FIGURE 3:**
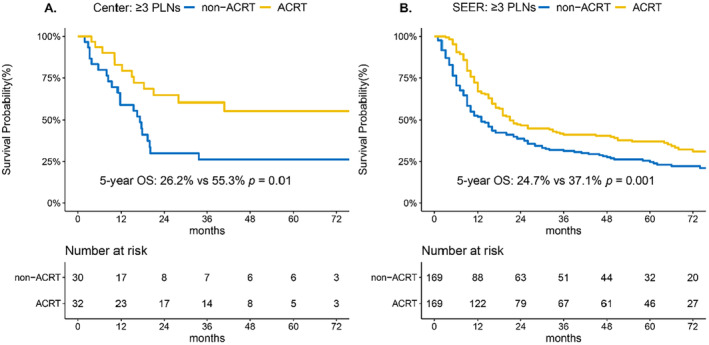
Kaplan–Meier curves of overall survival (OS) in patients with advanced nodal disease (≥ 3 PLNs) with and without ACRT. After PSM, ACRT significantly improved the OS of patients with ≥ 3 PLNs in (A) our center and (B) SEER registry cohort, respectively. ACRT, adjuvant chemoradiotherapy; PLN, positive lymph node; SEER, Surveillance, Epidemiology, and End Results Program.

Among the 334, 122, and 62 patients with 0 PLN, 1–2 PLNs, and ≥ 3 PLNs, respectively, 23 (6.9%), 41 (33.6%), and 32 (51.6%) patients received ACRT, respectively. Patients with 0 PLN, 1–2 PLNs and ≥ 3 PLNs with and without ACRT were matched on a 1:1 basis, with age (≤ 44 vs. > 44 years), sex, body mass index, betel nut chewing, diabetes, pT stage, and the continuous number of dissected and metastatic lymph nodes as pairing factors. Owing to the balanced distribution of these matching factors among patients with ≥ 3 PLNs with and without ACRT, patients with ACRT in our center were matched with 23 and 36 patients without ACRT for 0 and 1–2 PLNs disease, respectively. ACRT further improved the OS of patients with ≥ 3 PLNs (5‐year OS: 26.2% vs. 55.3%; *p* = 0.01; Figure 3A and Table S3). This trend was observed in the SEER study population, where separate survival curves were generated for the ACRT and non‐ACRT groups after PSM (5‐year OS: 24.7% vs. 37.1%; *p* = 0.001; Figure 3B and Figure S3 and Table S4). In contrast, there was no significant difference in OS between patients who received ACRT and those who did not receive ACRT with 0 PLN (Figure S4 and Tables S5 and S6) or with 1–2 PLNs (Figure S5 and Tables S7 and S8), either at the institutional level or in the SEER registry (all *p* > 0.05).

## 4 Discussion

In this institutional‐level retrospective study, we found that the number of PLNs is a robust, independent predictor for OS, surpassing other nodal features. Additionally, the derived nodal category for OTSCC showed improved prediction performance over the 8th edition AJCC N‐staging. Moreover, we found that patients with ≥ 3 PLNs could benefit from ACRT. The external validation of the proposed nodal category in the SEER registry offers the potential for a simplified, comparably effective nodal framework in oral subgenus prognostication.

One important finding of this study is that the presence of ENE, lower neck involvement, and laterality were no longer independent predictors of survival when the number of PLNs for OTSCC was considered. A multicenter study involving 3704 patients with oral cancers emphasized the greater importance of the number of PLNs than of contralateral lymph node involvement [22]. These findings also align with a later larger‐scale study conducted by Ho et al. [8], which highlights the value of PLNs over nodal size and laterality. In their proposed staging system for N0, N1 (1 PLN/ENE‐), N2 (1 PLN/ENE+ or 2 PLNs), N3a (3–7 PLNs), and N3b (≥ 8 PLNs), the role of ENE in risk stratification, primarily among singularly affected nodes, is noteworthy. Another study [23] yielded analogous results, suggesting that patients with OTSCC with ≥ 2 PLNs fare worse than those with a single positive node, irrespective of ENE status. Given the correlation between the increased number of metastatic lymph nodes and increased ENE incidence [24, 25], segregating ENE status from mild or extensive nodal burden may have limited utility in prognostic stratification.

Spreading through the lymphatic chain vertically or invading surrounding tissues horizontally is a manifestation of tumor cell invasiveness in different dimensions. An increased number of metastatic lymph nodes signifies the inability of the body's immune system to effectively prevent cancer cells from disseminating or metastasizing via the lymphatic route [26]. For the presence of ENE, some authors associated its adverse prognosis with hematogenous dissemination, where cancer cells migrate into surrounding soft tissues, enter the blood circulation, and ultimately increase the risk of distant metastasis [27, 28]. Other studies have indicated that deposits of isolated tumor cells beyond the nodal capsule, which may increase the risk of regional recurrence [29, 30]. However, the underlying mechanism for the superior prognostic value of the number of metastatic lymph nodes compared with the presence of ENE remains elusive and requires further detailed exploration. In this study, we confirmed the dominant impact of the number of PLNs and grouped patients with one to two PLNs into a single‐risk subgroup due to their similar prognoses.

In recent studies, alternative methods for classifying lymph node metastasis, such as the lymph node ratio (LNR) [31, 32] and log odds of positive lymph nodes (LODDS) [33], have been proposed as prognostic indicators in patients with oral cancers. While one study demonstrated that the PLN, LNR, and LODDS offer comparable prognostic value [34], another found that only the PLN remained an independent prognostic factor for OS in multivariate analysis [35]. These findings suggest that the PLN may serve as a more straightforward and effective measure of nodal burden than complex mathematical models.

In line with this, studies have identified key PLN thresholds, such as ≥ 5 PLNs in head and neck cancer [36], with Ho et al. [8] and Sinha et al. [9] further validating the prognostic significance of this threshold for risk stratification in oral cancer and p16+ oropharyngeal SCC, respectively. However, our study of OTSCC patients identified a lower cutoff at 3 PLNs, which aligns with the findings of Rajappa et al. [37], who proposed a cutoff of ≥ 3 PLNs for advanced N3 staging. Our proposed nodal classification, specific to the subsite of the oral cavity, offers a simplified nodal factor with fewer risk groups and clearer survival distinctions. In particular, current N‐staging considers only patients with ≥ 1 PLN for upstaging, and our nodal category broadens stratification with more nodes. In addition, given the similar prognoses of one PLN and two PLNs, we further downstage the 8th AJCC N2 with 1–2 PLNs to N1 and found an improved C‐index, which also provides solutions for optimizing current pN‐staging by refining the PLN threshold (1–2 nodes).

The current National Comprehensive Cancer Network (NCCN) guidelines recommend that adjuvant therapy be based on individual patient characteristics [38]. Zumsteg et al. [39] reported that in 7144 nonoropharyngeal head and neck SCCs from the National Cancer Database, increasing metastatic nodal burden was associated with increased benefit from postoperative adjuvant chemoradiation relative to radiotherapy alone, corresponding to nearly 8% and 23% absolute improvements in 3‐year OS for subgroup patients with 3–5 PLNs and ≥ 6 PLNs, respectively, over patients with 0–2 PLNs. In contrast, when the same data registry was used, Spiotto et al. [40] demonstrated that OTSCC with ≥ 2 PLNs was associated with a benefit from adjuvant systematic treatment compared with surgery plus postoperative radiation alone (3‐year OS: 67.5% vs. 57.1%; *p* = 0.01). In our study, which utilized a matching method for OTSCC, we observed a significant improvement in 5‐year OS in the proposed advanced nodal category (≥ 3 PLNs) at our institution and in the SEER registry. This finding supports the clinical practicality of this proposed simplified nodal scheme, which allows adjuvant chemoradiation to be administered individually.

Several limitations warrant acknowledgment. First, our institution lacks data on nodal size—a parameter whose prognostic value has diminished in previous studies and may thus have less impact on our analysis. Second, despite the advantages of the proposed nodal scheme, replacing the current pN stage with it would represent a significant adjustment. Nevertheless, we aimed to optimize AJCC N‐staging in the subsite of the head and neck by providing a more representative nodal feature rather than replacing the existing traditional staging system. Finally, patients with pN0 constituted more than half of the study population; these patients have heterogeneous prognoses, and the discovery of more biomarkers is needed to make generalizable predictions.

## 5 Conclusion

In summary, we found that the number of metastatic lymph nodes was the strongest factor for long‐term tumor control in patients with OTSCC. Patients with ≥ 3 PLNs derived greater relative and absolute improvements in OS from postoperative systematic treatment than patients with 0–2 PLNs did. Our study included an intricate analysis of clinicopathological variables, providing a new, simplified nodal schema with improved risk discrimination in OTSCC.

## Author Contributions

**Wenjie Huang:** conceptualization (lead), data curation (lead), investigation (lead), resources (equal), software (lead), visualization (lead), writing – original draft (lead), writing – review and editing (lead). **Yu Zhang:** conceptualization (equal), data curation (equal), investigation (equal), resources (equal), supervision (equal), writing – review and editing (equal). **Hao Li:** investigation (equal), software (equal), writing – original draft (equal), writing – review and editing (equal). **Zhiying Liang:** data curation (supporting), investigation (supporting), writing – original draft (equal), writing – review and editing (equal). **Shumin Zhou:** investigation (supporting), visualization (supporting), writing – review and editing (supporting). **Jie Pan:** investigation (supporting), software (equal), visualization (equal), writing – review and editing (equal). **Hui Xie:** investigation (supporting), software (equal), visualization (equal), writing – review and editing (equal). **Chao Luo:** investigation (supporting), writing – original draft (equal), writing – review and editing (supporting). **Shuqi Li:** investigation (supporting), writing – original draft (equal), writing – review and editing (supporting). **Guangying Ruan:** data curation (supporting), investigation (supporting), writing – review and editing (supporting). **Fei Ai:** investigation (supporting), project administration (equal), supervision (equal), writing – review and editing (supporting). **Yanfeng Chen:** conceptualization (equal), investigation (supporting), project administration (equal), supervision (lead), writing – review and editing (equal).

## Ethics Statement

This retrospective study received approval from the Clinical Research Ethics Committee of the Sun Yat‐Sen University Cancer Center (approval number B2019‐047‐Y02).

## Consent

Informed consent was waived due to the retrospective nature of the study.

## Conflicts of Interest

The authors declare no conflicts of interest.

## Supporting information


Data S1.

## Data Availability

The data described in this study have been deposited at the Sun Yat‐Sen University Cancer Center for future reference (www.researchdata.org.cn) and are available upon reasonable request.
