# Supplementary material for: Prognostic Implications of the Number of Lymph Node Metastases in Oral Tongue Squamous Cell Carcinoma: A Population Study of the SEER Database and an Institutional Registry
Source: Cancer Med. 2024 Dec 19;13(24):e70493. doi: 10.1002/cam4.70493 (PMC11656219; doi:10.1002/cam4.70493)
Supplement: Supplementary file 1 — Data S1. [file CAM4-13-e70493-s001.docx]

**Table S1** Multivariate analyses of whole sample with OTSCC for selecting confounding factors of overall survival (N=518)

| **Variables** | **Multivariable cox (Enter）** | |  | **Multivariable cox (Stepwise）** | |
| --- | --- | --- | --- | --- | --- |
|  | **HR [95% CI]** | ***P*-value** |  | **HR [95% CI]** | ***P*-value** |
| Age | 1.02 (1.01, 1.04) | 0.007 |  | 1.02 (1, 1.04) | 0.010 |
| Smoking |  |  |  |  |  |
| No |  |  |  |  |  |
| Yes | 1.31 (0.89, 1.93) | 0.171 |  |  |  |
| Betel nut |  |  |  |  |  |
| No |  |  |  |  |  |
| Yes | 2.03 (1.09, 3.79) | 0.025 |  | 2.38 (1.31, 4.32) | 0.004 |
| Diabetes |  |  |  |  |  |
| No |  |  |  |  |  |
| Yes | 2.03 (1.19, 3.46) | 0.009 |  | 2.02 (1.19, 3.44) | 0.009 |
| Differentiation |  |  |  |  |  |
| No |  |  |  |  |  |
| Yes | 0.65 (0.44, 0.98) | 0.038 |  | 0.62 (0.42, 0.91) | 0.014 |
| Lymphovascular invasion | |  |  |  |  |
| No |  |  |  |  |  |
| Yes | 1.81 (0.9, 3.62) | 0.095 |  | 2.07 (1.06, 4.06) | 0.034 |
| Perineural invasion |  |  |  |  |  |
| No |  |  |  |  |  |
| Yes | 1.32 (0.89, 1.97) | 0.164 |  |  |  |
| ACRT |  |  |  |  |  |
| No |  |  |  |  |  |
| Yes | 1.07 (0.68, 1.69) | 0.772 |  |  |  |
| pT stage |  |  |  |  |  |
| T1 |  |  |  |  |  |
| T2 | 2.28 (1.25, 4.17) | 0.007 |  | 2.61 (1.46, 4.68) | 0.001 |
| T3 | 4.03 (2.21, 7.32) | 0.000 |  | 4.8 (2.76, 8.35) | 0.000 |
| T4 | 3.93 (1.27, 12.19) | 0.018 |  | 4.54 (1.49, 13.84) | 0.008 |

Note. —The non-nodal-related clinicopathological variables with a P-value < 0.05 in univariate analysis were further included in multivariate analysis for the determination of confounding factors.

Abbreviations: OTSCC, oral tongue squamous cell carcinoma; HR, hazard ratio; CI, confidence interval; ACRT, adjuvant chemoradiotherapy.

**Table S2** Multivariate analyses of comparing the prognostic value of all nodal characteristics in patients with OTSCC regarding overall survival

|  | **Variables** | **Multivariable cox (Enter）** | |  | **Multivariable cox (Stepwise）** | |
| --- | --- | --- | --- | --- | --- | --- |
|  |  | **HR [95% CI]** | ***P*-value** |  | **HR [95% CI]** | ***P*-value** |
| **Nodal characteristics** | No. of dissected lymph nodes | 0.98 (0.96, 1) | 0.033 |  | 0.98 (0.96, 1) | 0.040 |
|  | No. of positive lymph nodes | 1.34 (1.21, 1.48) | <0.001 |  | 1.36 (1.26, 1.47) | <0.001 |
|  | Laterality |  |  |  |  |  |
|  | Unilaterality | 1 (reference) |  |  | Not included | |
|  | Bilaterality | 1.95 (0.56, 6.76) | 0.295 |  |  |  |
|  | ENE |  |  |  |  |  |
|  | Negative | 1 (reference) |  |  | Not included | |
|  | Positive | 1.4 (0.66, 2.96) | 0.382 |  |  |  |
|  | Lower neck involvement |  |  |  |  |  |
|  | No | 1 (reference) |  |  | Not included | |
|  | Yes | 1.03 (0.45, 2.34) | 0.945 |  |  |  |
| **Confounding factors** | Age | 1.02 (1.01, 1.04) | 0.006 |  | 1.02 (1.01, 1.04) | 0.003 |
|  | Betel nut |  |  |  |  |  |
|  | No | 1 (reference) |  |  | 1 (reference) |  |
|  | Yes | 2.94 (1.6, 5.42) | 0.001 |  | 2.91 (1.59, 5.32) | 0.001 |
|  | Diabetes |  |  |  |  |  |
|  | No | 1 (reference) |  |  | 1 (reference) |  |
|  | Yes | 2.25 (1.31, 3.88) | 0.003 |  | 2.19 (1.28, 3.75) | 0.004 |
|  | Lymphovascular involvement |  |  |  |  |  |
|  | No | 1 (reference) |  |  | 1 (reference) |  |
|  | Yes | 2.43 (1.22, 4.86) | 0.012 |  | 2.49 (1.26, 4.9) | 0.008 |
|  | Differentiation |  |  |  |  |  |
|  | Well | 1 (reference) |  |  | 1 (reference) |  |
|  | Moderate-poor | 0.76 (0.51, 1.12) | 0.162 |  | 0.74 (0.5, 1.1) | 0.134 |
|  | pT stage |  |  |  |  |  |
|  | T1 | 1 (reference) |  |  | 1 (reference) |  |
|  | T2 | 2.52 (1.39, 4.54) | 0.002 |  | 2.48 (1.38, 4.48) | 0.002 |
|  | T3 | 3.67 (2.06, 6.52) | <0.001 |  | 3.64 (2.05, 6.45) | <0.001 |
|  | T4 | 1.85 (0.56, 6.15) | 0.315 |  | 1.97 (0.61, 6.4) | 0.257 |

Abbreviations: OTSCC, oral tongue squamous cell carcinoma; ENE, extranodal extension; HR, hazard ratio; CI, confidence interval.

**Table S3** Clinicopathological characteristics of patients with OTSCC and ≥3 PLNs who received ACRT in our center dataset

|  | **Non-ACRT** | **ACRT** | ***P*-value** |
| --- | --- | --- | --- |
|  | **(N=30)** | **(N=32)** |  |
| Age (y) |  |  |  |
| ≤44 | 6 (20 %) | 14 (44 %) | 0.084 |
| >44 | 24 (80 %) | 18 (56 %) |  |
| Sex |  |  |  |
| Male | 20 (67 %) | 25 (78 %) | 0.468 |
| Female | 10 (33 %) | 7 (22 %) |  |
| BMI |  |  |  |
| Mean ± SD | 23.4 ± 3.60 | 22.2 ± 3.43 | 0.183 |
| Median [Min, Max] | 23.6 [15.6, 31.2] | 21.9 [17.3, 31.6] |  |
| Diabetes |  |  |  |
| No | 28 (93 %) | 29 (91 %) | 1 |
| Yes | 2 (7 %) | 3 (9 %) |  |
| Betel nut |  |  |  |
| No | 29 (97 %) | 28 (88 %) | 0.391 |
| Yes | 1 (3 %) | 4 (12 %) |  |
| No. of positive lymph nodes | | |  |
| Mean ± SD | 4.43 ± 2.28 | 4.66 ± 2.79 | 0.731 |
| Median [Min, Max] | 3.00 [3.00, 12.0] | 4.00 [3.00, 18.0] |  |
| No. of dissected lymph nodes | | |  |
| Mean ± SD | 28.7 ± 18.2 | 26.0 ± 9.53 | 0.478 |
| Median [Min, Max] | 22.0 [10.0, 89.0] | 23.5 [11.0, 46.0] |  |
| T stage |  |  |  |
| T1 | 2 (7 %) | 2 (6 %) | 0.933 |
| T2 | 10 (33 %) | 9 (28 %) |  |
| T3 | 17 (57 %) | 19 (59 %) |  |
| T4 | 1 (3 %) | 2 (6 %) |  |
| N stage |  |  |  |
| N0 | 0 (0 %) | 0 (0 %) | 1.000 |
| N1 | 0 (0 %) | 0 (0 %) |  |
| N2 | 23 (77 %) | 24 (75 %) |  |
| N3 | 7 (23 %) | 8 (25 %) |  |
| deadtime |  |  |  |
| Mean ± SD | 26.5 ± 28.6 | 32.4 ± 26.0 | 0.398 |
| Median [Min, Max] | 16.8 [1.90, 105] | 25.6 [3.57, 104] |  |

Abbreviations: PLN, positive lymph node; OTSCC, oral tongue squamous cell carcinoma; ACRT, adjuvant chemoradiotherapy; BMI, body mass index; NA, not available.

**Table S4** Clinicopathological characteristics of patients with OTSCC and ≥3 PLNs who received ACRT before and after PSM in the SEER dataset

|  | **Before PSM** | | |  | **After PSM** | | |
| --- | --- | --- | --- | --- | --- | --- | --- |
|  | **Non-ACRT** | **ACRT** | ***P*-value** |  | **Non-ACRT** | **ACRT** | ***P*-value** |
|  | **(N=170)** | **(N=335)** |  |  | **(N=169)** | **(N=169)** |  |
| Age (y) |  |  |  |  |  |  |  |
| ≤44 | 21 (12 %) | 52 (16 %) | 0.41 |  | 21 (12 %) | 30 (18 %) | 0.224 |
| >44 | 149 (88 %) | 283 (84 %) |  |  | 148 (88 %) | 139 (82 %) |  |
| Sex |  |  |  |  |  |  |  |
| Female | 59 (35 %) | 114 (34 %) | 0.958 |  | 58 (34 %) | 57 (34 %) | 1 |
| Male | 111 (65 %) | 221 (66 %) |  |  | 111 (66 %) | 112 (66 %) |  |
| Differentiation | |  |  |  |  |  |  |
| Well | 21 (12 %) | 28 (8 %) | 0.203 |  | 20 (12 %) | 20 (12 %) | 1 |
| Moderate-poor | 149 (88 %) | 307 (92 %) |  |  | 149 (88 %) | 149 (88 %) |  |
| No. of positive lymph nodes | | |  |  |  |  |  |
| Mean ± SD | 5.58 ± 5.67 | 6.01 ± 5.44 | 0.415 |  | 5.59 ± 5.68 | 5.56 ± 4.06 | 0.956 |
| Median [Min, Max] | 4.00 [3.00, 51.0] | 4.00 [3.00, 69.0] | |  | 4.00 [3.00, 51.0] | 4.00 [3.00, 31.0] | |
| No. of dissected lymph nodes | | |  |  |  |  |  |
| Mean ± SD | 40.6 ± 19.9 | 44.7 ± 22.5 | 0.035 |  | 40.7 ± 19.9 | 42.0 ± 20.4 | 0.533 |
| Median [Min, Max] | 39.0 [12.0, 90.0] | 41.0 [10.0, 90.0] | |  | 39.0 [12.0, 90.0] | 40.0 [10.0, 90.0] | |
| T stage |  |  |  |  |  |  |  |
| T1 | 26 (15 %) | 53 (16 %) | 0.241 |  | 25 (15 %) | 27 (16 %) | 0.925 |
| T2 | 69 (41 %) | 114 (34 %) |  |  | 69 (41 %) | 66 (39 %) |  |
| T3 | 45 (26 %) | 84 (25 %) |  |  | 45 (27 %) | 49 (29 %) |  |
| Tx | 30 (18 %) | 84 (25 %) |  |  | 30 (18 %) | 27 (16 %) |  |
| N stage |  |  |  |  |  |  |  |
| N0 | 0 (0 %) | 0 (0 %) | 0.035 |  | 0 (0 %) | 0 (0 %) | 0.186 |
| N1 | 27 (16 %) | 30 (9 %) |  |  | 27 (16 %) | 16 (9 %) |  |
| N2 | 141 (83 %) | 295 (88 %) |  |  | 140 (83 %) | 150 (89 %) |  |
| N3 | 2 (1 %) | 10 (3 %) |  |  | 2 (1 %) | 3 (2 %) |  |
| deadtime |  |  |  |  |  |  |  |
| Mean ± SD | 28.8 ± 31.2 | 35.1 ± 30.9 | 0.032 |  | 28.4 ± 30.9 | 36.3 ± 30.4 | 0.018 |
| Median [Min, Max] | 13.0 [1.00, 119] | 19.0 [3.00, 116] | |  | 13.0 [1.00, 119] | 21.0 [3.00, 111] | |

Abbreviations: PLN, positive lymph node; OTSCC, oral tongue squamous cell carcinoma; ACRT, adjuvant chemoradiotherapy; PSM, propensity score matching; SEER, Surveillance, Epidemiology, and End Results Program; NA, not available.

**Table S5** Clinicopathological characteristics of patients with OTSCC and 0 PLNs who received ACRT before and after PSM in center dataset

|  | **Before PSM** | | |  | **After PSM** | | |
| --- | --- | --- | --- | --- | --- | --- | --- |
|  | **Non-ACRT** | **ACRT** | ***P*-value** |  | **Non-ACRT** | **ACRT** | ***P*-value** |
|  | **(N=311)** | **(N=23)** |  |  | **(N=23)** | **(N=23)** |  |
| Age (y) |  |  |  |  |  |  |  |
| ≤44 | 87 (28 %) | 8 (35 %) | 0.646 |  | 6 (26 %) | 8 (35 %) | 0.749 |
| >44 | 224 (72 %) | 15 (65 %) |  |  | 17 (74 %) | 15 (65 %) |  |
| Sex |  |  |  |  |  |  |  |
| Male | 197 (63 %) | 20 (87 %) | 0.039 |  | 20 (87 %) | 20 (87 %) | 1 |
| Female | 114 (37 %) | 3 (13 %) |  |  | 3 (13 %) | 3 (13 %) |  |
| BMI |  |  |  |  |  |  |  |
| Mean ± SD | 22.9 ± 3.15 | 22.5 ± 3.35 | 0.591 |  | 22.7 ± 3.84 | 22.5 ± 3.35 | 0.886 |
| Median [Min, Max] | 22.5 [15.8, 34.6] | 22.0 [18.0, 33.4] | |  | 22.1 [17.1, 31.9] | 22.0 [18.0, 33.4] | |
| Diabetes |  |  |  |  |  |  |  |
| No | 282 (91 %) | 21 (91 %) | 1 |  | 23 (100 %) | 21 (91 %) | 0.47 |
| Yes | 29 (9 %) | 2 (9 %) |  |  | 0 (0 %) | 2 (9 %) |  |
| Betel nut |  |  |  |  |  |  |  |
| No | 293 (94 %) | 21 (91 %) | 0.911 |  | 21 (91 %) | 21 (91 %) | 1 |
| Yes | 18 (6 %) | 2 (9 %) |  |  | 2 (9 %) | 2 (9 %) |  |
| No. of dissected lymph nodes | | |  |  |  |  |  |
| Mean ± SD | 20.1 ± 7.81 | 21.6 ± 8.45 | 0.436 |  | 24.3 ± 9.73 | 21.6 ± 8.45 | 0.314 |
| Median [Min, Max] | 18.0 [10.0, 58.0] | 23.0 [12.0, 49.0] | |  | 22.0 [12.0, 50.0] | 23.0 [12.0, 49.0] | |
| T stage |  |  |  |  |  |  |  |
| T1 | 136 (44 %) | 1 (4 %) | <0.001 |  | 1 (4 %) | 1 (4 %) | 1 |
| T2 | 111 (36 %) | 10 (43 %) |  |  | 10 (43 %) | 10 (43 %) |  |
| T3 | 61 (20 %) | 11 (48 %) |  |  | 11 (48 %) | 11 (48 %) |  |
| T4 | 3 (1 %) | 1 (4 %) |  |  | 1 (4 %) | 1 (4 %) |  |
| N stage |  |  |  |  |  |  |  |
| N0 | 311 (100 %) | 23 (100 %) | NA |  | 23 (100 %) | 23 (100 %) | NA |
| N1 | 0 (0 %) | 0 (0 %) |  |  | 0 (0 %) | 0 (0 %) |  |
| N2 | 0 (0 %) | 0 (0 %) |  |  | 0 (0 %) | 0 (0 %) |  |
| N3 | 0 (0 %) | 0 (0 %) |  |  | 0 (0 %) | 0 (0 %) |  |
| deadtime |  |  |  |  |  |  |  |
| Mean ± SD | 46.7 ± 26.3 | 38.2 ± 24.1 | 0.114 |  | 31.5 ± 15.0 | 38.2 ± 24.1 | 0.268 |
| Median [Min, Max] | 41.4 [2.87, 115] | 32.4 [6.47, 103] | |  | 29.9 [7.47, 67.0] | 32.4 [6.47, 103] | |

Abbreviations: PLN, positive lymph node; OTSCC, oral tongue squamous cell carcinoma; ACRT, adjuvant chemoradiotherapy; PSM, propensity score matching; BMI, body mass index; NA, not available.

**Table S6** Clinicopathological characteristics of patients with OTSCC and 0 PLNs who received ACRT before and after PSM in the SEER dataset

|  | **Before PSM** | | |  | **After PSM** | | |
| --- | --- | --- | --- | --- | --- | --- | --- |
|  | **Non-ACRT** | **ACRT** | ***P*-value** |  | **Non-ACRT** | **ACRT** | ***P*-value** |
|  | **(N=1253)** | **(N=68)** |  |  | **(N=68)** | **(N=68)** |  |
| Age (y) |  |  |  |  |  |  |  |
| ≤44 | 185 (15 %) | 17 (25 %) | 0.035 |  | 12 (18 %) | 17 (25 %) | 0.402 |
| >44 | 1068 (85 %) | 51 (75 %) |  |  | 56 (82 %) | 51 (75 %) |  |
| Differentiation | |  |  |  |  |  |  |
| Well | 322 (26 %) | 7 (10 %) | 0.007 |  | 7 (10 %) | 7 (10 %) | 1 |
| Moderate-poor | 931 (74 %) | 61 (90 %) |  |  | 61 (90 %) | 61 (90 %) |  |
| Sex |  |  |  |  |  |  |  |
| Female | 523 (42 %) | 27 (40 %) | 0.838 |  | 28 (41 %) | 27 (40 %) | 1 |
| Male | 730 (58 %) | 41 (60 %) |  |  | 40 (59 %) | 41 (60 %) |  |
| No. of dissected lymph nodes | | |  |  |  |  |  |
| Mean ± SD | 31.3 ± 16.1 | 34.6 ± 18.0 | 0.136 |  | 35.9 ± 19.3 | 34.6 ± 18.0 | 0.69 |
| Median [Min, Max] | 28.0 [10.0, 90.0] | 30.0 [11.0, 86.0] | |  | 32.0 [10.0, 90.0] | 30.0 [11.0, 86.0] | |
| T stage |  |  |  |  |  |  |  |
| T1 | 697 (56 %) | 14 (21 %) | <0.001 |  | 14 (21 %) | 14 (21 %) | 1 |
| T2 | 424 (34 %) | 27 (40 %) |  |  | 27 (40 %) | 27 (40 %) |  |
| T3 | 91 (7 %) | 12 (18 %) |  |  | 12 (18 %) | 12 (18 %) |  |
| Tx | 41 (3 %) | 15 (22 %) |  |  | 15 (22 %) | 15 (22 %) |  |
| N stage |  |  |  |  |  |  |  |
| N0 | 1249 (100 %) | 68 (100 %) | NA |  | 68 (100 %) | 68 (100 %) | NA |
| N1 | 2 (0 %) | 0 (0 %) |  |  | 0 (0 %) | 0 (0 %) |  |
| N2 | 2 (0 %) | 0 (0 %) |  |  | 0 (0 %) | 0 (0 %) |  |
| N3 | 0 (0 %) | 0 (0 %) |  |  | 0 (0 %) | 0 (0 %) |  |
| deadtime |  |  |  |  |  |  |  |
| Mean ± SD | 66.0 ± 29.6 | 61.3 ± 32.7 | 0.251 |  | 56.9 ± 32.2 | 61.3 ± 32.7 | 0.429 |
| Median [Min, Max] | 66.0 [1.00, 119] | 66.0 [3.00, 117] | |  | 56.0 [1.00, 119] | 66.0 [3.00, 117] | |

Abbreviations: PLN, positive lymph node; OTSCC, oral tongue squamous cell carcinoma; ACRT, adjuvant chemoradiotherapy; PSM, propensity score matching; SEER, Surveillance, Epidemiology, and End Results Program; NA, not available.

**Table S7** Clinicopathological characteristics of patients with OTSCC and 1-2 PLNs who received ACRT before and after PSM in our center dataset

|  | **Before PSM** | | |  | **After PSM** | | |
| --- | --- | --- | --- | --- | --- | --- | --- |
|  | **Non-ACRT** | **ACRT** | ***P*-value** |  | **Non-ACRT** | **ACRT** | ***P*-value** |
|  | **(N=81)** | **(N=41)** |  |  | **(N=32)** | **(N=32)** |  |
| Age (y) |  |  |  |  |  |  |  |
| ≤44 | 18 (22 %) | 15 (37 %) | 0.141 |  | 9 (28 %) | 10 (31 %) | 1 |
| >44 | 63 (78 %) | 26 (63 %) |  |  | 23 (72 %) | 22 (69 %) |  |
| Sex |  |  |  |  |  |  |  |
| Male | 59 (73 %) | 28 (68 %) | 0.755 |  | 22 (69 %) | 22 (69 %) | 1 |
| Female | 22 (27 %) | 13 (32 %) |  |  | 10 (31 %) | 10 (31 %) |  |
| BMI |  |  |  |  |  |  |  |
| Mean ± SD | 22.3 ± 3.57 | 23.8 ± 3.16 | 0.022 |  | 24.0 ± 3.53 | 23.1 ± 2.58 | 0.226 |
| Median [Min, Max] | 22.3 [15.2, 33.5] | 23.5 [18.3, 33.2] |  |  | 24.0 [18.2, 33.5] | 23.2 [18.3, 27.8] | |
| Diabetes |  |  |  |  |  |  |  |
| No | 71 (88 %) | 40 (98 %) | 0.142 |  | 31 (97 %) | 31 (97 %) | 1 |
| Yes | 10 (12 %) | 1 (2 %) |  |  | 1 (3 %) | 1 (3 %) |  |
| Betel nut |  |  |  |  |  |  |  |
| No | 76 (94 %) | 37 (90 %) | 0.727 |  | 31 (97 %) | 29 (91 %) | 0.606 |
| Yes | 5 (6 %) | 4 (10 %) |  |  | 1 (3 %) | 3 (9 %) |  |
| No. of positive lymph nodes | | |  |  |  |  |  |
| Mean ± SD | 1.49 ± 0.503 | 1.59 ± 0.499 | 0.343 |  | 1.50 ± 0.508 | 1.59 ± 0.499 | 0.459 |
| Median [Min, Max] | 1.00 [1.00, 2.00] | 2.00 [1.00, 2.00] |  |  | 1.50 [1.00, 2.00] | 2.00 [1.00, 2.00] | |
| No. of dissected lymph nodes | | |  |  |  |  |  |
| Mean ± SD | 23.3 ± 10.3 | 25.8 ± 10.1 | 0.204 |  | 23.7 ± 9.58 | 25.3 ± 9.09 | 0.481 |
| Median [Min, Max] | 21.0 [10.0, 66.0] | 24.0 [11.0, 48.0] |  |  | 21.5 [10.0, 49.0] | 24.5 [13.0, 46.0] | |
| T stage |  |  |  |  |  |  |  |
| T1 | 10 (12 %) | 5 (12 %) | 0.071 |  | 4 (12 %) | 5 (16 %) | 0.574 |
| T2 | 37 (46 %) | 11 (27 %) |  |  | 13 (41 %) | 9 (28 %) |  |
| T3 | 31 (38 %) | 25 (61 %) |  |  | 15 (47 %) | 18 (56 %) |  |
| T4 | 3 (4 %) | 0 (0 %) |  |  | 0 (0 %) | 0 (0 %) |  |
| N stage |  |  |  |  |  |  |  |
| N0 | 0 (0 %) | 0 (0 %) | 0.525 |  | 0 (0 %) | 0 (0 %) | 0.606 |
| N1 | 40 (49 %) | 17 (41 %) |  |  | 16 (50 %) | 13 (41 %) |  |
| N2 | 38 (47 %) | 21 (51 %) |  |  | 15 (47 %) | 16 (50 %) |  |
| N3 | 3 (4 %) | 3 (7 %) |  |  | 1 (3 %) | 3 (9 %) |  |
| deadtime |  |  |  |  |  |  |  |
| Mean ± SD | 40.1 ± 27.7 | 29.4 ± 18.2 | 0.012 |  | 33.3 ± 21.0 | 29.7 ± 17.5 | 0.46 |
| Median [Min, Max] | 35.6 [3.30, 114] | 26.9 [5.53, 83.9] |  |  | 34.6 [4.63, 87.5] | 27.7 [5.53, 83.9] | |

Abbreviations: PLN, positive lymph node; OTSCC, oral tongue squamous cell carcinoma; ACRT, adjuvant chemoradiotherapy; PSM, propensity score matching; BMI, body mass index; NA, not available.

**Table S8** Clinicopathological characteristics of patients with OTSCC and 1-2 PLNs who received ACRT before and after PSM in the SEER dataset

|  | **Before PSM** | | |  | **After PSM** | | |
| --- | --- | --- | --- | --- | --- | --- | --- |
|  | **Non-ACRT** | **ACRT** | ***P*-value** |  | **Non-ACRT** | **ACRT** | ***P*-value** |
|  | **(N=478)** | **(N=269)** |  |  | **(N=248)** | **(N=248)** |  |
| Age (y) |  |  |  |  |  |  |  |
| ≤44 | 54 (11 %) | 52 (19 %) | 0.004 |  | 38 (15 %) | 39 (16 %) | 1 |
| >44 | 424 (89 %) | 217 (81 %) |  |  | 210 (85 %) | 209 (84 %) |  |
| Differentiation | |  |  |  |  |  |  |
| Well | 55 (12 %) | 24 (9 %) | 0.328 |  | 33 (13 %) | 22 (9 %) | 0.153 |
| Moderate-poor | 423 (88 %) | 245 (91 %) |  |  | 215 (87 %) | 226 (91 %) |  |
| Sex |  |  |  |  |  |  |  |
| Female | 199 (42 %) | 103 (38 %) | 0.415 |  | 93 (38 %) | 96 (39 %) | 0.853 |
| Male | 279 (58 %) | 166 (62 %) |  |  | 155 (62 %) | 152 (61 %) |  |
| No. of positive lymph nodes | | |  |  |  |  |  |
| Mean ± SD | 1.31 ± 0.461 | 1.51 ± 0.501 | <0.001 |  | 1.46 ± 0.499 | 1.48 ± 0.500 | 0.72 |
| Median [Min, Max] | 1.00 [1.00, 2.00] | 2.00 [1.00, 2.00] | |  | 1.00 [1.00, 2.00] | 1.00 [1.00, 2.00] | |
| No. of dissected lymph nodes | | |  |  |  |  |  |
| Mean ± SD | 32.4 ± 17.4 | 35.5 ± 19.1 | 0.027 |  | 33.9 ± 18.5 | 36.1 ± 19.1 | 0.19 |
| Median [Min, Max] | 29.0 [10.0, 90.0] | 30.0 [10.0, 90.0] | |  | 30.0 [10.0, 90.0] | 31.0 [10.0, 90.0] | |
| T stage |  |  |  |  |  |  |  |
| T1 | 173 (36 %) | 66 (25 %) | <0.001 |  | 58 (23 %) | 64 (26 %) | 0.902 |
| T2 | 212 (44 %) | 114 (42 %) |  |  | 115 (46 %) | 110 (44 %) |  |
| T3 | 60 (13 %) | 55 (20 %) |  |  | 46 (19 %) | 43 (17 %) |  |
| Tx | 33 (7 %) | 34 (13 %) |  |  | 29 (12 %) | 31 (12 %) |  |
| N stage |  |  |  |  |  |  |  |
| N0 | 0 (0 %) | 0 (0 %) | <0.001 |  | 0 (0 %) | 0 (0 %) | 0.194 |
| N1 | 341 (71 %) | 132 (49 %) |  |  | 147 (59 %) | 127 (51 %) |  |
| N2 | 133 (28 %) | 133 (49 %) |  |  | 98 (40 %) | 117 (47 %) |  |
| N3 | 4 (1 %) | 4 (1 %) |  |  | 3 (1 %) | 4 (2 %) |  |
| deadtime |  |  |  |  |  |  |  |
| Mean ± SD | 51.4 ± 32.9 | 51.5 ± 34.8 | 0.962 |  | 49.1 ± 34.0 | 52.3 ± 34.8 | 0.295 |
| Median [Min, Max] | 53.0 [1.00, 119] | 55.0 [4.00, 117] | |  | 50.5 [1.00, 119] | 56.0 [4.00, 117] | |

Abbreviations: OTSCC, oral tongue squamous cell carcinoma; ACRT, adjuvant chemoradiotherapy; PSM, propensity score matching; SEER, Surveillance, Epidemiology, and End Results Program; NA, not available.

**Figure S1**

**
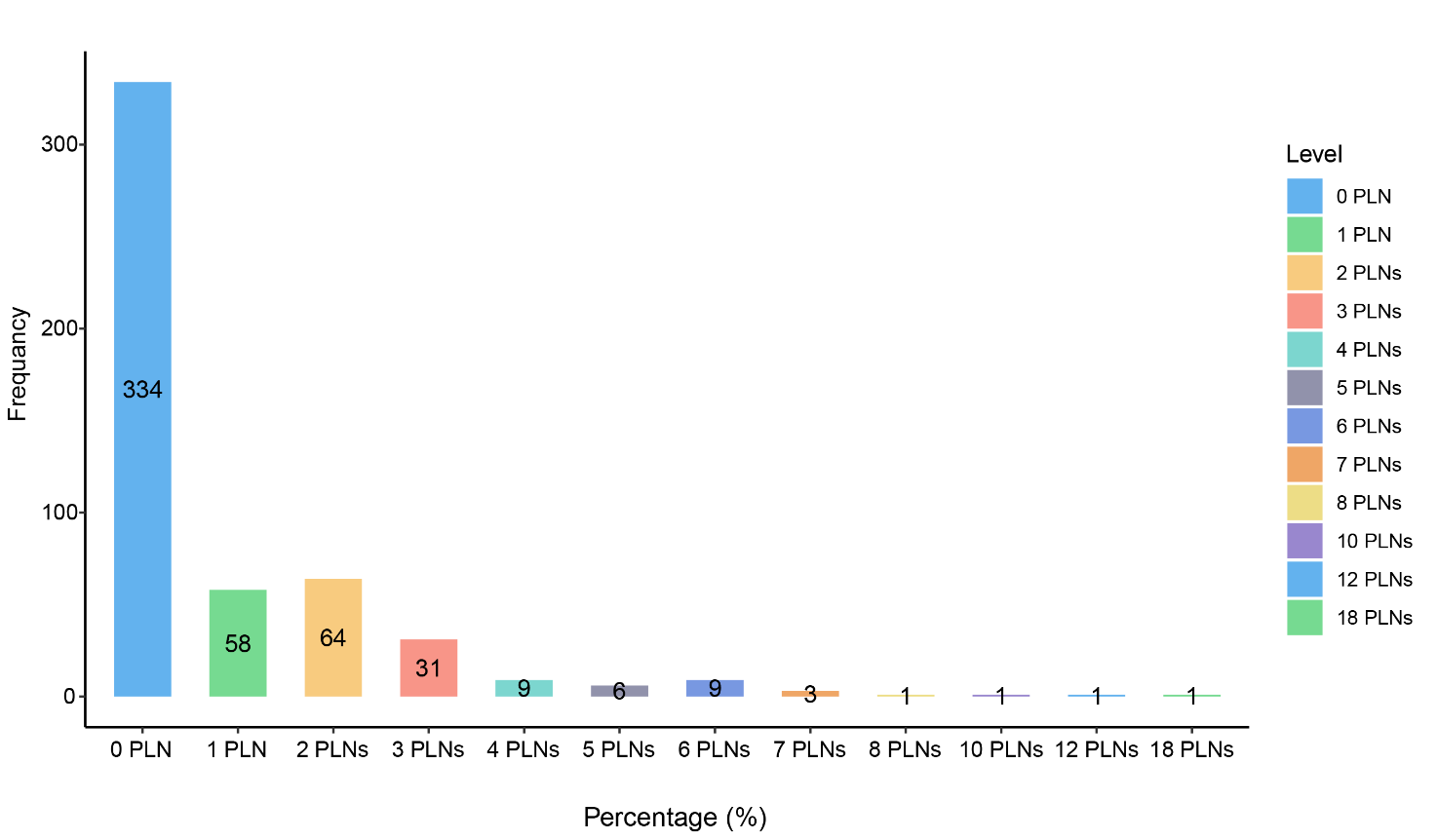
Figure S1** The distribution of the PLN.

Abbreviation: PLN, positive lymph node.

**Figure S2**


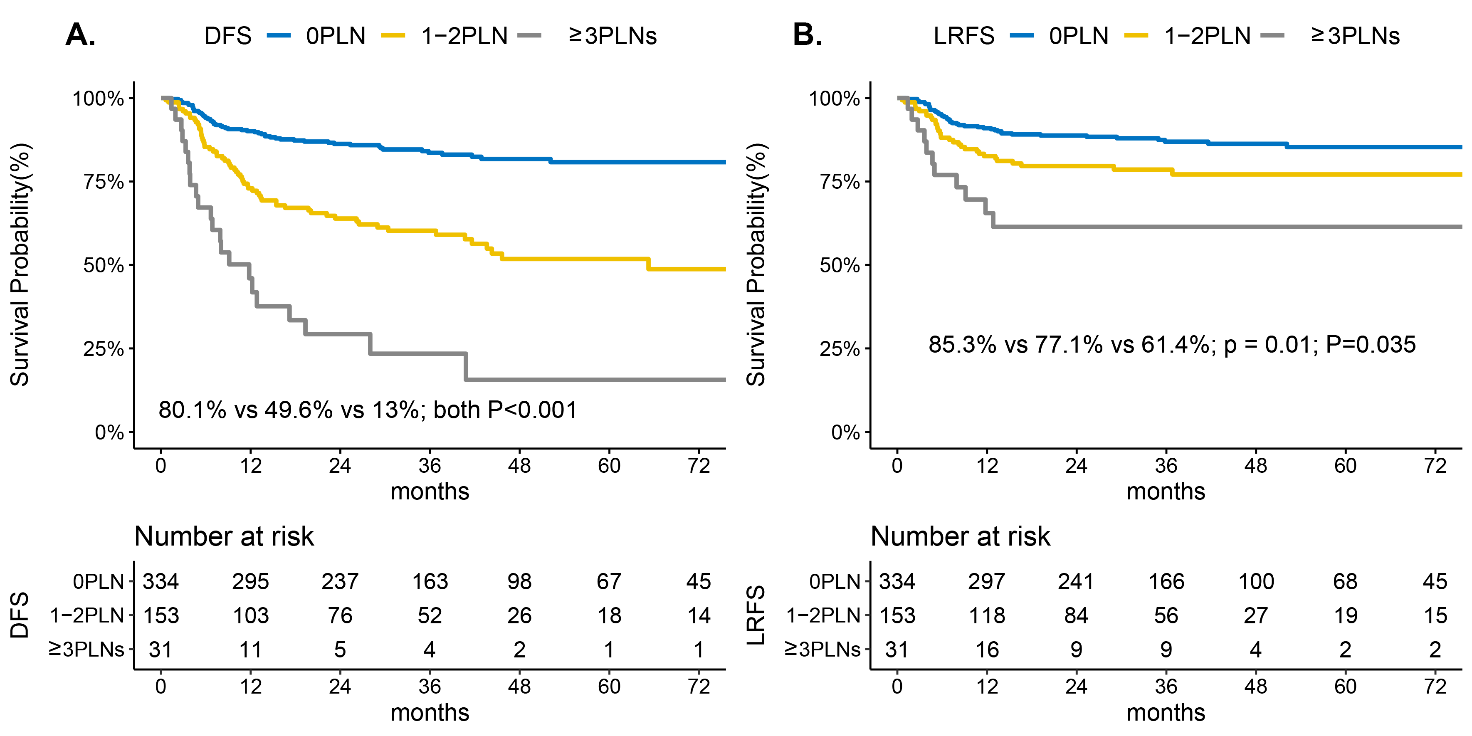


**Figure S2** Kaplan-Meier analyses of DFS and LRFS among patients who had no nodal disease, 1-2 PLNs and ≥3 PLNs.

Abbreviation: PLN, positive lymph node; DFS, disease-free survival; LRFS, locoregional-free survival.

**Figure S3**

**
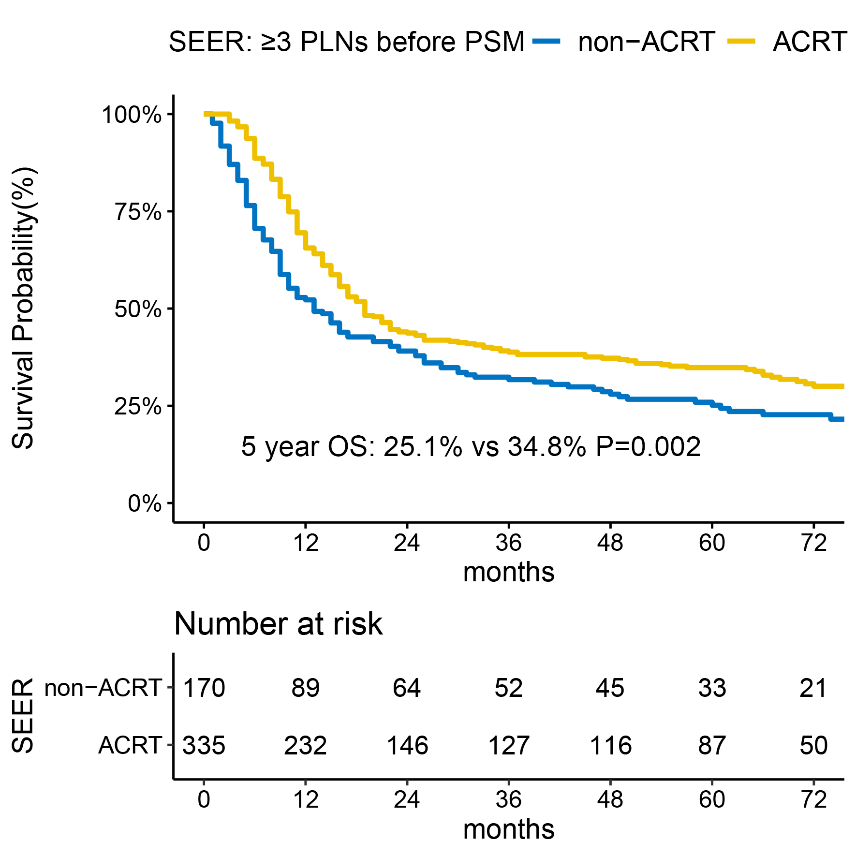
**

**Figure S3** Kaplan-Meier analyses of OS in patients with ≥3 PLNs receiving ACRT and not receiving ACRT before PSM in SEER registry cohort.

Abbreviation: PLN, positive lymph node; OS, overall survival; SEER, Surveillance, Epidemiology, and End Results Program; ACRT, adjuvant chemoradiotherapy; PSM, propensity score matching.

**Figure S4**


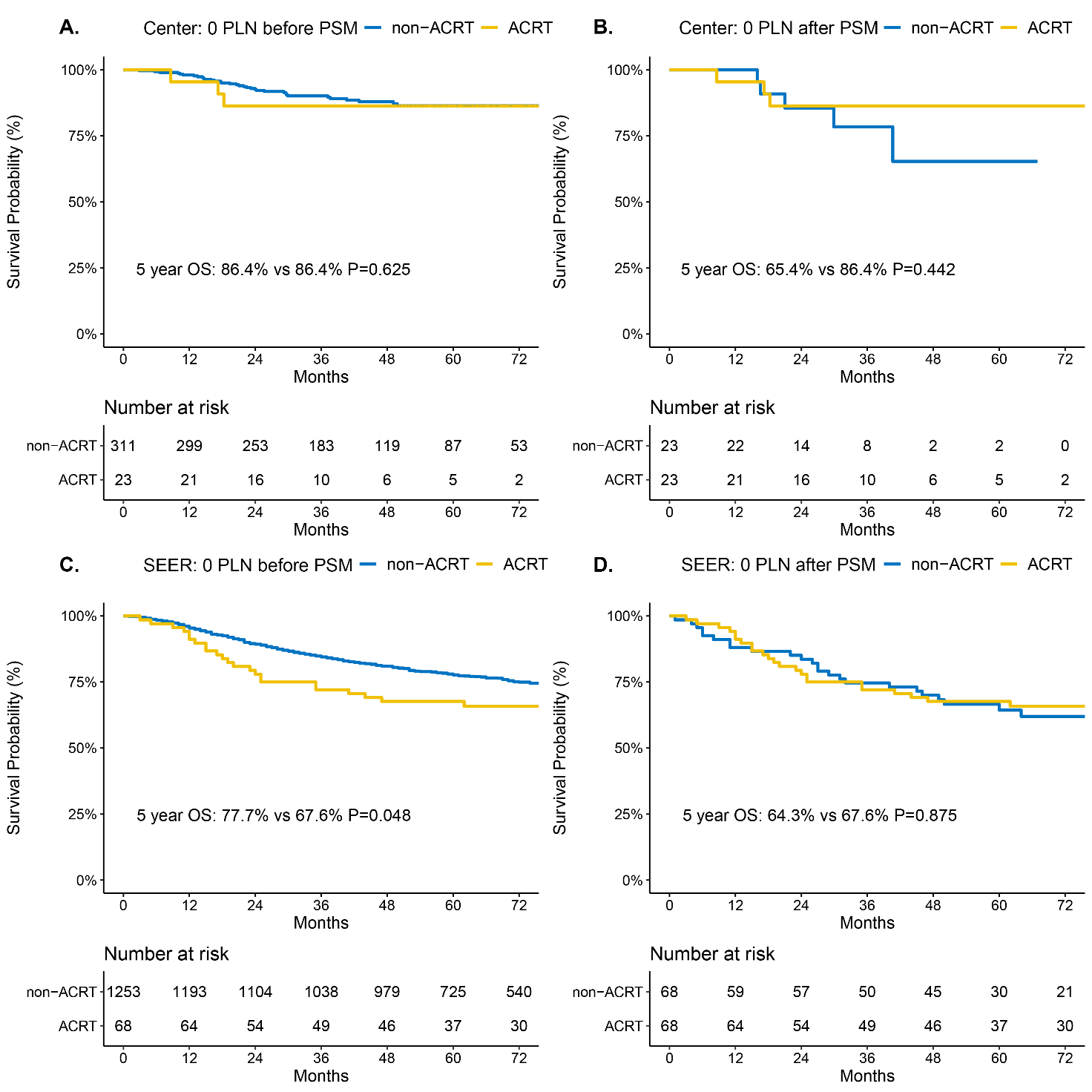


**Figure S4** Kaplan-Meier analyses of OS in patients staged as 0 PLN with and without ACRT after curative resection.

Abbreviations: PLN, positive lymph node; ACRT, adjuvant chemoradiotherapy; SEER, Surveillance, Epidemiology, and End Results Program; OS, overall survival; PSM, propensity score matching.

**Figure S5**

**
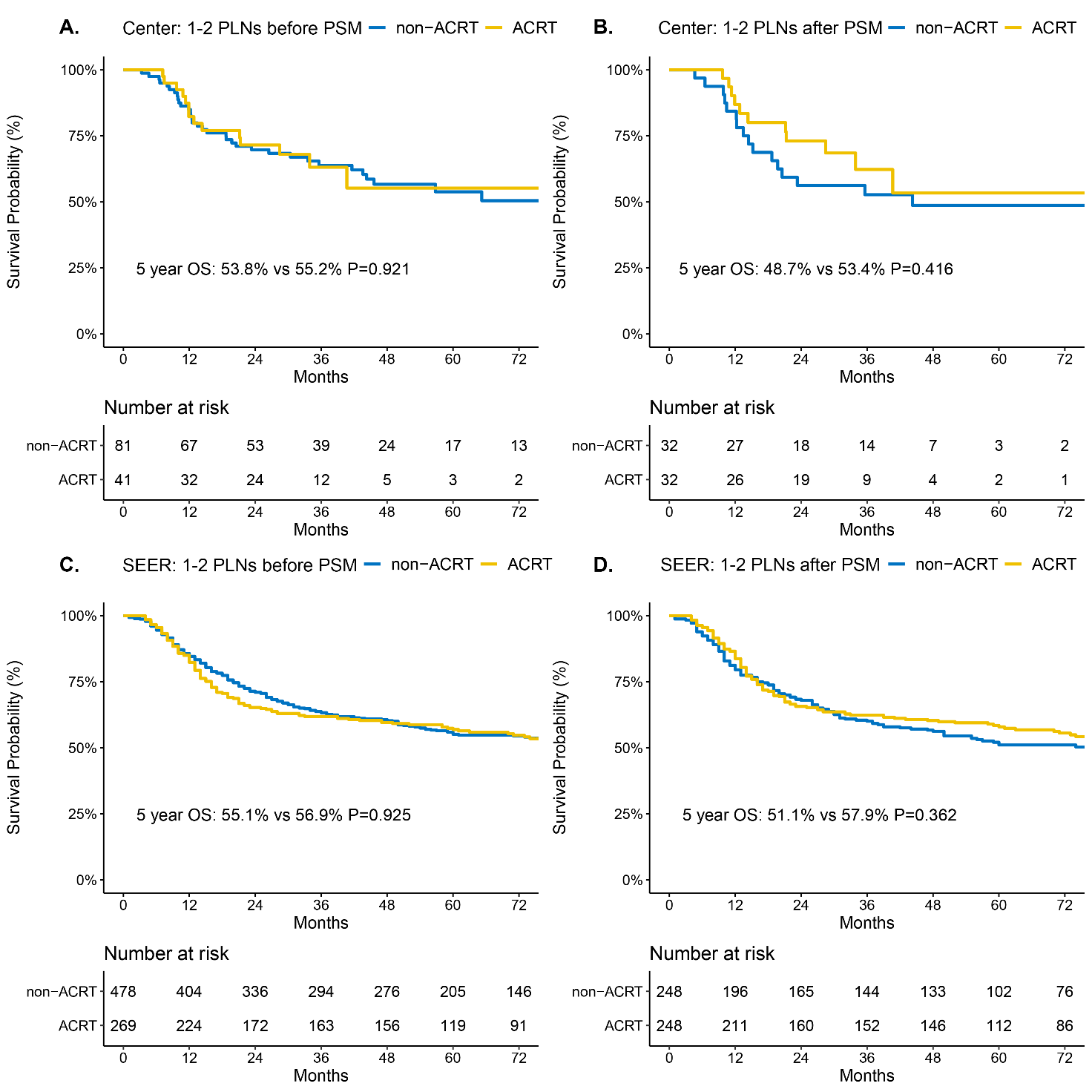
**

**Figure S5** Kaplan-Meier analyses of OS in patients with 1-2 PLNs receiving ACRT and not receiving ACRT after curative resection.

Abbreviations: PLN, positive lymph node; ACRT, adjuvant chemoradiotherapy; SEER, Surveillance, Epidemiology, and End Results Program; OS, overall survival; PSM, propensity score matching.

**Adjuvant treatment for patients with OTSCC**

Patients with adverse pathological characteristics were recommended to receive adjuvant radiotherapy or adjuvant chemotherapy under the National Comprehensive Cancer Network guidelines,^1^ including pT3/4, pN2/3, extranodal extension, positive margin, perineural and lymphovascular involvement and lower (level IV or V) neck involvement. A total of 127 of 518 (24.5%) patients received cisplatin-based chemotherapy consisting of 60 mg/m2 cisplatin plus 260 mg/m2 paclitaxel weekly or 80-100 mg/m2 cisplatin on days 1, 22 and 43 of radiotherapy. Target volumes were delineated slice by slice on the treatment-planning CT scans using an individualized delineation protocol in accordance with International Commission on Radiation Units and Measurements reports 62 and 83. The gross tumor volumes (GTVs), including the primary tumor (GTVnx) and involved cervical lymph nodes (GTVnd), were determined from the imaging findings at presentation. The clinical target volume in the high-risk regions (CTV-1) included the GTVnx with a 5-15 mm margin (if possible) and the whole superficial oral tongue. ^2^ The CTV for the low-risk regions (CTV-2) covered all at-risk uninvolved nodal levels. The planning target volumes (PTVs) were determined from the GTVs or CTVs, with a 3-5 mm margin for setup variations. The radiation doses for each target volume were as follows: 66-72 Gy in 30-33 fractions to the PTV of GTVnx; 64–70 Gy to the PTV of GTVnd; 60-66 Gy to the PTV of CTV-1; and 54-62 Gy to the PTV of CTV-2. Salvage treatment for recurrent and refractory cases included surgery, reirradiation, and chemotherapy.

1. National Comprehensive Cancer Network. NCCN clinical practice guidelines in oncology- head and neck cancers [M]; Version1 ed. Pennsylvania: NCCN; 2023.
2. Evans M, Beasley M. Target delineation for postoperative treatment of head and neck cancer. Oral Oncol. 2018 Nov; 86:288-295.
